# Risk‐based prioritization of organic substances in the Canadian National Pollutant Release Inventory using an evaluative regional‐scale multimedia mass balance model

**DOI:** 10.1002/ieam.4601

**Published:** 2022-04-05

**Authors:** Alicia Berthiaume, Jon A. Arnot, Liisa Toose

**Affiliations:** ^1^ Science and Risk Assessment Directorate Environment and Climate Change Canada Gatineau Quebec Canada; ^2^ ARC Arnot Research and Consulting Inc. Toronto Ontario Canada; ^3^ Department of Physical and Environmental Sciences University of Toronto Scarborough Toronto Ontario Canada; ^4^ Department of Pharmacology and Toxicology University of Toronto Toronto Ontario Canada

**Keywords:** Canada, National Pollutant Release Inventory (NPRI), Pollutant emissions, Risk Assessment IDentification And Ranking (RAIDAR)

## Abstract

The National Pollutant Release Inventory (NPRI) is a Canadian inventory of facility‐reported data on releases, transfers, and disposals of over 300 pollutants, but it does not contain information on chemical properties or other characteristics critical to understanding environmental and human health risks. To reconcile this gap, we use the Risk Assessment IDentification And Ranking (RAIDAR) model to integrate NPRI release data with chemical property information in a multimedia mass balance model to combine exposure estimates with toxicity hazard data yielding an estimate of risk for 198 NPRI organic substances reported in 2010–2019. The presented case study further corroborates the hypothesis that risk‐based ranking gives rise to different chemical priorities versus ranking based on release quantity alone. Chemicals like propane and hexane (except *n*‐hexane) are in the top 10 highest‐ranked organic substances based on emission quantities reported to NPRI but are ranked outside the top 10 based on corresponding regional‐scale risk estimates. On the contrary, dioxins and furans are ranked very low based on emissions quantities reported to NPRI but are ranked higher based on corresponding risk estimates. The results also suggest that although quantities of some NPRI organic pollutant releases change over time, the ensuing risk estimates are not always directly proportional to these changes. This can be explained by changes in mode of entry to the environment that can influence the overall fate and exposure of the same chemicals, highlighting the complex dynamics that can occur when simulating fate and risk as opposed to quantity alone. Limitations are discussed and recommendations are provided for improving the priority setting methods, including reducing the uncertainty of the NPRI data and the need for multimedia models to address point source emissions. *Integr Environ Assess Manag* 2022;18:1722–1732. © 2022 The Authors. *Integrated Environmental Assessment and Management* published by Wiley Periodicals LLC on behalf of Society of Environmental Toxicology & Chemistry (SETAC).

## KEY POINTS

- The NPRI is a Canadian inventory of pollutant releases, transfers, and disposals, but does not contain chemical property information or other characteristics critical to understanding environmental and human health risk.
- Here, we use the RAIDAR model to integrate NPRI release data with chemical property information in a multimedia mass balance model to combine exposure estimates with toxicity hazard data yielding an estimate of risk for certain NPRI organic substances.
- The results show that risk‐based ranking of NPRI substances gives different priorities than ranking based on release quantity alone, and that while pollutant releases change over time, the ensuing risk estimates are not always directly proportional to these changes.
- Limitations and recommendations are discussed for improving the approach.

## INTRODUCTION

The primary objective of chemical regulatory programs is to estimate, and if necessary, mitigate the potential risk of chemicals released to the environment (Government of Canada [GOC], 2016a). The Canadian Environmental Protection Act, 1999, SC 1999, c 33 (CEPA) is a key mechanism by which chemicals are assessed and managed to protect the environment and well‐being of Canadians. The National Pollutant Release Inventory (NPRI) is the Canadian Pollutant Release and Transfer Register (PRTR) legislated under CEPA to provide a publicly accessible inventory of pollutant releases (also called emissions to the environment) (GOC, 2021a). The NPRI program was established in 1993 to address the growing public interest in the nature and quantity of chemicals released in Canada that could potentially adversely impact human health and the environment (Environment Canada, 1992). The “community‐right‐to‐know” driver for the NPRI stemmed from the similar context and evolution of the United States Toxic Release Inventory (US TRI) established several years earlier (1986) (United States Environmental Protection Agency [US EPA], 2020), and continues to drive PRTRs around the world (Organisation for Economic Cooperation and Development [OECD], 2019). The NPRI is one of the multiple mechanisms that help identify candidates for chemical risk assessment in Canada (GOC, 2020).

However, as discussed by Lifset (2001) and Dunn (2009), among others, chemical release quantity alone (e.g., mass) is a poor proxy for environmental and/or human health risk, which is a function of not only exposure but also of hazard (OECD, 2019). Indeed, there is a growing global shift in PRTRs to move beyond the simple provision of data on the quantity of chemicals being released, to more contextual information on progress toward human health and environmental risk management and sustainability objectives (OECD, 2019). For example, the United Nations Global Round Table on PRTRs called for the next generation of PRTRs to provide knowledge‐on‐demand; specifically, the presentation of PRTR data with other datasets for a variety of purposes such as risk ranking, hazard ranking, exposure assessment, and guidance or tools to use PRTR data to assess risk or exposure (OECD, 2019). Doing so in consideration of both human and ecological organisms will also advance our understanding of planetary health and the (local to global scale) ability to evaluate progress toward sustainability, particularly toward the UN Sustainable Development Goal 12.4: the sound management of chemicals and waste by significantly reducing their releases to air, water, and soil in order to minimize impacts on human health and the environment (OECD, 2019).

In response, PRTRs around the world are increasingly providing products or tools that address the call to integrate pollutant release data into more holistic interpretations of risk, impact, and priorities. Among the many examples, Japan's Organisation for Research and Communication on Environmental Risk of Chemicals relates pollutant release data to pollutant risk by translating local level emissions into estimated atmospheric concentrations and compares these values to the associated risk‐based target management value for environmental quality (National Institute of Advance Industrial Science and Technology, 2005). The US EPA is associated with two options to characterize risk based on PRTR emissions. The Risk‐Screening Environmental Indicators combine American PRTR data from the US TRI with human (but not environmental) exposure modeling and chemical‐specific toxicity weighting (Langlois et al., 2018). The Tool for Reduction and Assessment of Chemicals and Other Environmental Impacts is a lifecycle impact assessment tool that can combine US TRI data with other characterization factors (e.g., ecotoxicity, human toxicity, and various impacts to the environment upon which life depends) to achieve a relative ranking of environmental and human health impacts (Bare, 2011). The USEtox model, endorsed by the United Nations Environment Programme and the Society of Environmental Toxicology and Chemistry (Hauschild et al., 2008), has been used to interpret and rank data from the European Pollutant Release and Transfer Register (E‐PRTR) in terms of potential human and environmental impacts of pollutants in Sweden and Europe (European Environmental Agency, 2018; Nordborg et al., 2017).

In Canada, chemical‐specific information, such as toxicity, bioaccumulation, environmental fate, and transport properties required for such holistic interpretations of exposure and risk, are not currently captured directly in the NPRI. Thus, there is a need to gather and interpret this chemical property information outside the inventory to achieve a more holistic view of NPRI data in terms of risk, as is being called for internationally. One approach is to parameterize and apply multimedia environmental fate and exposure model(s) for NPRI substances, and then use NPRI release data in such model(s) to quantify the relationship between releases, exposure estimates, and potential risk. While analyses that aim to better understand a chemical's impact by combining environmental releases with toxicity and exposure properties have been conducted on subsets of NPRI data (e.g., by Dunn, 2009) using the CHEMS‐1 model, and by Taylor et al. (2020) using the USEtox 2.0 model, these previous approaches have certain limitations. For example, the CHEMS‐1 study did not estimate exposure based on fate and transport properties of a substance, but rather weighted substances on a scale (of 1–10) relative to the highest quantity released per environmental compartment and thus only provides a risk ranking relative to other substances in the analysis as opposed to an absolute risk estimate (Dunn, 2009). The Taylor et al. (2020) study is limited by the geographic scope of the study (Nova Scotia), and the scope of the model used. Most notably, USEtox is a lifecycle assessment tool providing relative rankings of risk (as opposed to absolute risk characterization) and does not consider toxicokinetic processes, such as absorption, distribution, biotransformation, and excretion, in a range of receptors (Mitchell et al., 2013).

The objective of this work is to build on past efforts and to demonstrate proof of concept for using multimedia fate and exposure models to better quantify relationships between national organic chemical releases as reported in the NPRI database and potential risks to a diverse range of ecological receptors and humans. We use Risk Assessment IDentification And Ranking (RAIDAR), a multimedia mass balance model for organic substances, which was developed to quantify and evaluate chemical exposures and potential risks to humans and ecological receptors (Arnot & Mackay, 2008; Arnot et al., 2006). The RAIDAR model has been used in various contexts (e.g., Arnot et al., 2012; Liu et al., 2021; Shin et al., 2015), including high throughput risk‐based screening and regulatory initiatives in Canada (Bonnell et al., 2018). We first develop a database of chemical information required to parameterize environmental fate and exposure models for organic chemicals on the NPRI (see Supporting Information for parameterization details). We use NPRI release data to estimate emission rates and conduct RAIDAR (version 2.999442b) model simulations at a regional‐scale using a default environment typical of temperate Canada (Arnot et al., 2006). The model results for exposure and risk estimation are used to rank and prioritize chemicals on the NPRI list. We explore, compare, and discuss the outputs of the different approaches used here to rank chemicals either by emission quantity (rate) or by risk. Recommendations for future directions on the topic of combining release data, such as NPRI data with models for fate, exposure, and risk are provided.

## DATA AND METHODS

### Overview of the NPRI data

The NPRI comprises measured or estimated data reported by qualifying point‐source facilities (e.g., in general, facilities with 10 employees or more who handle amounts of listed substances above key thresholds, though there are exceptions) on releases to air, water, and land; transfers for treatment, recycling, or disposal; and disposals for over 300 substances (Environment and Climate Change Canada [ECCC], 2019). A full list of NPRI substances and their reporting thresholds can be found on the NPRI website (GOC, 2021a) and descriptions of the release categories are available in Table S1. There are multiple accepted methods that facilities may use to calculate data they report to the NPRI (i.e., continuous emission monitoring systems, predictive emission monitoring, source testing, mass balance, site‐specific emission factors, published emission factors, and engineering estimates) and their use varies by sector and by the substance being reported depending on technology and information availability and other regulatory contexts (ECCC, 2019). The release data discussed herein were most often estimated based on published emission factors or engineering estimates. The data are mainly reported as annual totals per facility per substance but are also broken down further in some cases (e.g., monthly or quarterly totals, subtotals by stack or electricity generating unit, average effluent concentrations, etc.). Notably, this level of reporting does not adequately describe all release fluctuations throughout the year. The collected data are made publicly available on Canada's open data portal and on the NPRI website in both interpretive information products (e.g., fact sheets), and in various data products (e.g., spreadsheets or databases). The interested reader is encouraged to explore NPRI data on the NPRI website (GOC, 2021a). The 2010–2019 data used in the current approach were extracted from the raw disaggregated release table on the GOC open data portal (https://open.canada.ca/data/en/dataset/40e01423-7728-429c-ac9d-2954385ccdfb) and comprised of annual totals by facility and environmental compartment for NPRI substances. Case studies in the Discussion section aggregated these data in space, time, and by industrial sector.

#### Considerations regarding the NPRI

While the NPRI provides a substantial view of chemical point‐source releases in Canada, there are known limitations in its ability to represent the full picture of pollution sources in Canada and these are presented upfront here to delineate the context of this study. For example, the scope and mandate of the NPRI are to capture releases of certain substances of human health or ecological concern from medium and large anthropogenic point sources (e.g., mainly facilities with 10 or more employees) in Canada. However, NPRI generally excludes potentially significant small and/or noncommercial point‐sources, diffuse or area sources, and nonanthropogenic sources as well as substances that are not listed on the NPRI, such as those that are not in commerce or are prohibited in Canada (GOC, 2021b). As well, errors, noncompliance in reporting by some qualifying facilities, and/or uncertainty in the data stemming from the various acceptable calculation methods used by facilities to report to the NPRI (SENES Consulting Limited, 2011) may also leave some chemical inputs unaccounted for, or over/underestimated. More fulsome discussions on NPRI limitations are found in GOC (2021b), Berthiaume (2021), and Johnston Edwards & Walker (2020). Nonetheless, the NPRI contributes important perspectives on medium or large point‐sources of pollutants in Canada and thus is pivotal in the national prioritization of chemicals for risk assessment and/or risk management, which is the focus of this proof of concept study.

### Overview of the RAIDAR model

The RAIDAR model combines mechanistic mass balance environmental fate and food web bioaccumulation models to quantify chemical transport from *diffuse sources* in an evaluative, regional‐scale environment to representative ecological receptors and humans. (Arnot & Mackay, 2008; Arnot et al., 2006;  Arnot et al., 2012). RAIDAR can include hazard data to combine with the exposure estimates to calculate the likelihood of human health and/or environmental risk. The RAIDAR model requires a minimal number of chemical‐specific input parameters that can be obtained from empirical databases or quantitative structure–activity relationships (QSARs). The model has been peer‐reviewed and used in various application contexts (e.g., Mitchell et al., 2013; Ring et al., 2019) including previous chemical assessments conducted by the GOC (ECCC, 2016). RAIDAR is publicly available (www.eas-e-suite.com), thus facilitating transparency for use in regulatory programs. A conceptual description of RAIDAR is detailed below and in the Supporting Information; equations and default parameters of the model are available in Arnot and Mackay (2008) and Arnot et al. (2010).

In the current application, RAIDAR calculates chemical fate in the environment (air, water, soil, sediment, and vegetation) using Level III (steady‐state, nonequilibrium) mass balance equations. Level III model simulations require information about chemical mode of entry (MOE) to the environment, that is, chemical emissions to air, water, or soil or some combination of the three. The default evaluative environment used in the current application includes a regional landscape with an area of 100 000km^2^ (90% land, 10% water with underlying sediment). The “default” geographical conditions of the physical environment are not specific to any region and are like those of the EQuilibrium Criterion model (Mackay et al., 1996) that are representative of temperate North America. RAIDAR calculates environmental persistence (i.e., average residence time of the chemical in the environmental medium (Mackay & Webster, 2006) and percentage chemical distribution in environmental compartments. All reactions are treated as first‐order based on user‐selected, chemical‐specific half‐lives in air, water, soil, and sediment. Rates of transport between compartments are estimated using typical environmental transport parameters, such as precipitation rates and mass transfer coefficients. A substance can be removed from the regional environment by degradation at rates characterized by the reaction half‐lives, and by advective losses (flows of air and water leaving the environment, and by burial in sediments). In this study, we assume that chemical outflow from an adjacent region does not flow into the simulated environment.

A variety of receptor organisms are included in RAIDAR, such as representative plant, invertebrate, and vertebrate species, including fish and wildlife (mammals and birds of different feeding strategies), agricultural crops and livestock, and an adult human (Arnot & Mackay, 2008). The bioaccumulation submodels calculate concentrations in the representative species based on concentrations in their environment and their diets. Bioaccumulation for vertebrates is calculated using species‐specific one‐compartment physiologically based toxicokinetic models. Chemical‐specific uptake processes in the bioaccumulation models include respiration and food and water ingestion. Chemical‐specific elimination processes in the bioaccumulation models include respiratory loss, fecal egestion, and biotransformation. Urinary excretion is included for air‐respiring organisms. Reproductive losses are also included for certain species of relevance for human exposures, for example, reproductive (calving) and lactation loss processes for dairy cattle and reproductive losses for egg‐laying hens. Active transport processes of uptake and elimination are assumed negligible compared to passive diffusion. For each representative organism, typical rates of feeding, respiration, egestion, and growth are assumed and estimated chemical absorption efficiencies are included.

The RAIDAR model calculates concentrations in the environment and ecological and human receptors using the fugacity approach (Mackay, 2001). Exposure intake rates are also calculated. These estimated exposures can be compared against toxicity hazard thresholds, such as effect or no effect concentrations (for ecological risk) or the Threshold of Toxicological Concern (for human risks) (Health Canada, 2016). For chemical comparisons, ranking, and priority setting objectives, RAIDAR also calculates the Exposure Assessment Factor (EAF), the Hazard Assessment Factor (HAF), and the Risk Assessment Factor (RAF) for each chemical of interest (Arnot & Mackay, 2008). As summarized in Table 1, the EAF combines aspects of chemical fate (persistence [P] and multimedia distribution) and food web bioaccumulation (B) in the defined environment. The EAF essentially quantifies the capacity of the environment to deliver a chemical to a receptor after it is released. Higher EAFs reflect higher exposure *potential* independent of actual chemical emission rates. The HAF combines the EAF with toxicity (T) data also independent of actual emission rates. Thus, in the absence of chemical use and release information, chemicals can be compared and prioritized based on their combined “PBT” hazards using the HAF metric. The risk characterization ratio (RCR) or Risk Quotient (RQ) is the ratio of the exposure estimate (i.e., using estimates of actual emission rates) to the selected toxicity hazard threshold (e.g., no effects level or LC50) for each representative species in the modeled environment. The RAF is determined for each chemical in the evaluative environment and is determined from the highest RCR calculated for each organism. In other words, while RAIDAR calculates exposure, combined hazard, and risk for each chemical and each organism, the EAF, HAF, and RAF represent the highest value for each chemical. In this manner, the model provides screening‐level risk estimates so that chemicals of interest can be compared based on the likelihood of adverse effects. The difference between HAF and RAF is that HAF uses the same emission rate for all chemicals. When emission rates are available, the RAF can be used for risk‐based chemical screening and priority setting. Further details of these metrics are provided elsewhere (Arnot & Mackay, 2008).

**Table 1:** Summary of the various RAIDAR (Risk Assessment IDentification And Ranking) model outputs

	Exposure assessment factor (EAF)	Hazard assessment factor (HAF)	Risk assessment factor	Risk characterization ratio
Description	Combines aspects of chemical fate (persistence [P], multimedia distribution) and food web bioaccumulation (B) in the defined environment. No emissions characterization	Combines the EAF with toxicity (T) data, such as effect or no effect information (e.g., Lethal (or Effect) Concentration 50 (L[E]C50) with a defined effect endpoint or threshold, *C*_T_; mmol/L, or an intake rate threshold, *IR*_T_; mg/kg/day)	Combines HAF with actual emission rates	Combines HAF with actual emission rates
		It is a combined “PBT” metric. No emissions characterization		
Emission scenario	Unit emission rate (1 kg/h) for all chemicals	Unit emission rate (1 kg/h) for all chemicals	Actual emission rates for all chemicals	Actual emission rates for all chemicals
Characterization	The highest exposure potential (i.e., in which compartment within the evaluative environment) per substance, *independent of actual emissions*	The highest risk potential in the most sensitive receptor organism in the evaluative environment, per substance, *independent of actual emissions*	The highest risk in the most sensitive receptor in the evaluative environment per substance, *using actual emission rates*	The risk characterization for each receptor organism, per substance, *using actual emission rates*

#### Considerations regarding RAIDAR

As with the NPRI, there are several known considerations regarding the application of RAIDAR in the context of this study, and these are presented here (up front) to further delineate the scope of this analysis as a proof of concept effort with future opportunities for refinement (expanded on in the Discussion section). For example, as noted above, the temporal (steady‐state, assuming diffuse emissions) aspect of RAIDAR is not perfectly aligned with NPRI's point‐source emissions data, and the near‐field dynamics radiating from point‐sources are not accounted for, potentially underestimating temporal and geographical concentration gradients, and, thus, potential risk proximal to sources (Bonnell et al., 2018). The spatial (regional‐scale) aspect of RAIDAR, while representative of temperate North American conditions, does not currently address regional or site‐specific environmental characteristics, which may impact the model assumptions and dynamics (Arnot et al., 2006). Moreover, a well‐recognized challenge for fate model simulations is the general paucity of data and QSARs for estimating hydrolysis reaction half‐lives at a range of pH and temperature (Boethling et al., 2009; Khan et al., 2021). Hydrolysis half‐lives are model input parameters that were not considered in these simulations, which may have implications for the exposure and risk‐based ranking of chemicals that are subject to rapid hydrolysis in the environment (e.g., some isocyanates). As well, RAIDAR does not simulate the fate, exposure, and risk of inorganics, nor chemical disposals (e.g., NPRI substances reported as going to landfill, land application, underground injection, tailings, and waste rock, etc.), and so certain criteria air contaminants (CACs) (e.g., carbon monoxide, sulfur dioxide, nitrogen oxides, particulate matter), metals, and the NPRI data on disposals were not included in the analysis. Nonetheless, the suitability of the RAIDAR model was deemed sufficient for this proof of concept study to better understand NPRI data in the context of screening‐level prioritization of potential risks to a diverse range of ecological receptors and humans.

### Model input parameters for NPRI organic substances

Table 2 summarizes a new database of chemical information for 252 NPRI organic substances (GOC, 2016b) required as input parameters for the RAIDAR model simulations. As shown in Table 2, the 252 chemicals capture a broad range of chemical partitioning, degradation, and toxicity values. The 2010–2019 time span was chosen for subsequent analysis using actual emissions because this dataset was relatively stable in which NPRI reporting requirements for organic substances did not change significantly (GOC, 2021a). Actual emission rate data (i.e., *E*_A_, based on NPRI annual substance totals from across Canada, per substance for each of 2010–2019, scaled to kg/h) were available for only 198 of the 252. We further focus some of the results and discussion on 2019 release data only comprising 168 of the 252 NPRI chemicals. A further detailed explanation of the model input parameters and their derivation is available in the Supporting Information.

**Table 2:** Summary of the range and medians of model input parameters for the organic chemicals in the NPRI database (GOC, 2016a)

Model input parameter (units)	Range (and median) of input parameters
*M* (g/mol)	26.0 to 959.2 (134.8)
Log *K*_OW_ (dimensionless)	−3.8 to 12.1 (2.4)
Log *K*_AW_ (dimensionless)	−14.3 to 2.5 (−3.2)
HL‐Air (h)	0.19 to 10^6^ (36.6)
HL‐Water (h)	55 to 7.2 × 10^4^ (480)
HL‐Soil (h)	110 to 1.4 × 10^5^ (970)
HL‐Sediment (h)	494 to 6.5 × 10^5^ (4.4 × 10^3^)
HL_B_‐Invertebrates and fish (h) @ 0.01 kg	0.16 to 6.6 × 10^3^ (8.5)
HL_B_‐Mammals and birds (h) @ 70 kg	0.05 to 8.5 × 10^5^ (4.8)
*C*_Toxicity_‐Autotrophs (mmol/L) (*n* = 143)	1.2 × 10^−6^ to 230 (0.11)
*C*_Toxicity_‐Invertebrates (mmol/L) (*n* = 214)	1.4 × 10^−6^ to 1270 (0.12)
*C*_Toxicity_‐Fish (mmol/L) (*n* = 235)	3.1 × 10^−8^ to 997 (0.17)
*IR*_Threshold_‐Birds, mammals (mg/kg/day)	0.15
*E*_A_ (kg/h)^a^	1.14 × 10^−13^ to 1.5 × 10^3^ (9.4 × 10^−3^)

Abbreviations: *E*_A_, estimate of regional‐scale actual chemical emission rate; HL, half‐life; *K*_AW_, air–water partition coefficient; *K*_OW_, octanol–water partition coefficient; *M*, molar mass; NPRI, National Pollutant Release Inventory.

^a^ *E*_A_ is based on NPRI annual substance totals from across Canada for 2010–2019, scaled to kg/h.

## RESULTS AND DISCUSSION

### Comparison of different approaches to ranking results

**Figure 1:**
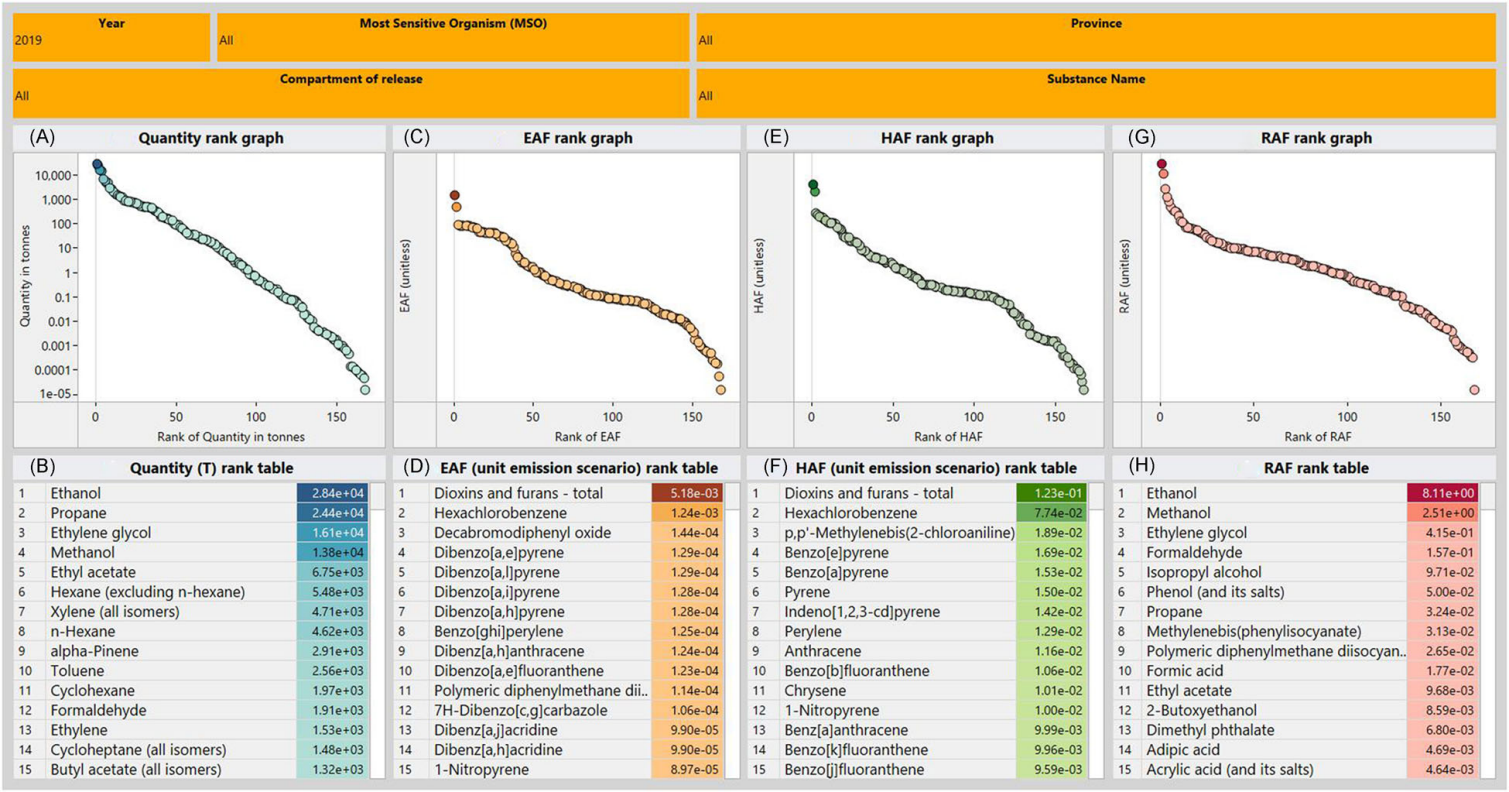
Summary of 2019 National Pollutant Release Inventory substances ranked by different approaches https://public.tableau.com/app/profile/alicia.berthiaume/viz/Figure1-SummaryofNPRISubstances2010-2019rankedbyQuantityTExposurepotentialHazardpotentialandRiskpotentialusingRAIDAR/MAINdashboard?publish=yes

The primary objective of this work is to characterize NPRI release data in exposure and risk‐based contexts by combining information on quantities and media of release, chemical properties, degradation rates, and toxicity, in a regional‐scale multimedia mass balance model for simulating their environmental fate, exposure, and potential risk to a diverse range of ecological receptors. To this end, Figure 1 summarizes the chemical ranking results from several different perspectives (i.e., the EAF, HAF, and RAF model calculations) for NPRI releases reported in 2019 using the RAIDAR model and the database of RAIDAR input parameters. Figure 1 emphasizes that there are different results in chemical ranking when comparing substances based on release estimates (i.e., the sum of releases per substance, reported by all qualifying facilities across Canada for 2019) (panes A and B), or the corresponding exposure (panes C and D), hazard (panes E and F), or risk estimates (panes G and H). An interactive dashboard version of Figure 1 (see link in the caption) shows the expanded results for NPRI data from 2010–2019. Of the 168 organic substances reported in 2019 and simulated in RAIDAR for this study, emission rates ranged from 10^−5^ to 28 kilotonnes/year. Ethanol, which is used as an industrial solvent, sanitizing agent, fuel, or as a component in beverages (Strohm, 2014), ranked highest (entirely released to air—the only compartment of release tracked by NPRI for this substance), and the group of dioxins and furans (total of 2,3,7,8‐tetrachlorodibenzodioxin [TCDD] toxic equivalents released via the burning of municipal wastes and fuels) ranked lowest based on quantity (mostly to air).

Concentrations in the environment are a function of the emission rates, conditions of the receiving environment, chemical distribution in the environment, and the relative rates of degradation within different environmental compartments (Boethling et al., 2009). The concept of overall persistence (*P*_OV_, reaction residence time) is well‐established, for example, by Scheringer et al. (2009). All else being equal, a chemical with a lower emission rate but higher persistence may have higher concentrations than a chemical with a higher emission rate that is rapidly degraded. This concept is highlighted in the current case study. The ranking order changed significantly when comparing the 2019 NPRI organic substances by relative exposure *potential* (i.e., EAF) calculated by the model (Figure 1C,D). Using an assumed unit emission rate of 1 kg/h for all chemicals (not actual emissions based on NPRI release data), exposure potential spans nine orders of magnitude. Whereas dioxins and furans ranked lowest by quantity released, these chemicals ranked highest by EAF. Dioxins and furans are recognized to be persistent and bioaccumulative in the environment. For example, 2,3,7,8‐TCDD (CAS# 1746‐01‐6) has an overall environmental persistence *P*_OV_ (reaction residence times calculated by RAIDAR *P*_OV_) of 600 days and a fish bioaccumulation factor of about 20 000 L/kg (US EPA, 2021). By contrast, whereas ethanol ranked highest by 2019 release quantity, this substance ranked in the bottom half (114^th^ of 168) based on EAF. Similarly, propane ranked second by quantity released but second last by EAF. Ethanol and propane are examples of chemicals with relatively low persistence and bioaccumulation in the environment, having an overall *P*_OV_ of 12 days and 20 days, respectively, an order of magnitude smaller than *P*_OV_ for 2,3,7,8‐TCDD. The interested reader can explore the dynamic data dashboard (link in the caption of Figure 1) to view the full breadth of changes and/or view results by an environmental compartment of release.

The rankings of NPRI substances change again based on the combined hazard potential (“PBT”) metric (HAF) (Figure 1E,F). As with the EAF, the HAF is based on an assumed unit emission rate of 1 kg/h for all chemicals; however, the HAF additionally includes toxicity and effects threshold data. The HAFs span six orders of magnitude for the 168 NPRI substances. While dioxins and furans were again the highest‐ranked substances based on HAFs, ethanol ranked in the top three, and propane ranked in the bottom quarter with an HAF four orders of magnitude lower than dioxins and furans.

Finally, when actual emission rates, determined from NPRI release quantities and MOE information for 2019, are combined with environmental exposure model estimates and selected hazard thresholds, the resulting RAIDAR‐calculated risk assessment factors (RAFs) (Figure 1G,H) change again. From this perspective, ethanol ranked 1^st^ (with the most sensitive organism being foliage vegetation for these air releases) and ranked dioxins and furans in the bottom third (140^th^), the most sensitive organism being a terrestrial carnivore. Propane, which ranked 2^nd^ by quantity release, ranked 7^th^ by risk relative to the other NPRI substances examined, with the small rodent being the most sensitive receptor organism for the air releases. More substantial shifts are noted for substances, such as methylenebis (phenylisocyanate), and polymeric diphenylmethane diisocyanate, which is used in the manufacture of polyurethanes and polyurethane products, such as adhesives, coatings, and insulation foams, among others. These substances moved from 50^th^ and 74^th^ rank by quantity to 8^th^ and 9^th^ rank by RAF, respectively, due to moderately high exposure and hazard potentials combined with moderately high quantities released. By contrast, xylene and *n*‐hexane are top 10 ranked substances by quantity but are ranked outside the top 10 based on risk. Note these NPRI release data are national‐scale values from multiple locations across Canada and are not contained within the 100 000 km^2^ regional‐scale hypothetical environment used in the RAIDAR evaluative environment nor have any scaling factors applied. This simplified approach was chosen for this proof of concept study on screening‐level NPRI substance prioritization; however, more precision could be introduced in future more geographically focused studies, as is discussed below in the section on further considerations. Thus, the results are primarily illustrative of the differences in ranking priorities when considering various levels of assessment and not actual risk, hazard, or exposure. Moreover, while the relative rankings are informative for determining priorities, it should also be noted that in a traditional RQ analysis context (e.g., comparison of exposure concentration to a selected endpoint concentration), the absolute value of the RAFs in this study were for the most part far less than 1. This suggests that the absolute magnitude of risk from any NPRI substance released in 2019 is not of significant concern, aside perhaps from the two highest priority cases identified here (ethanol and methanol, RAF > 1). More in‐depth assessment in these cases may be warranted to better understand the risk posed by these annual releases.

### Different approaches to ranking

**Figure 2:**
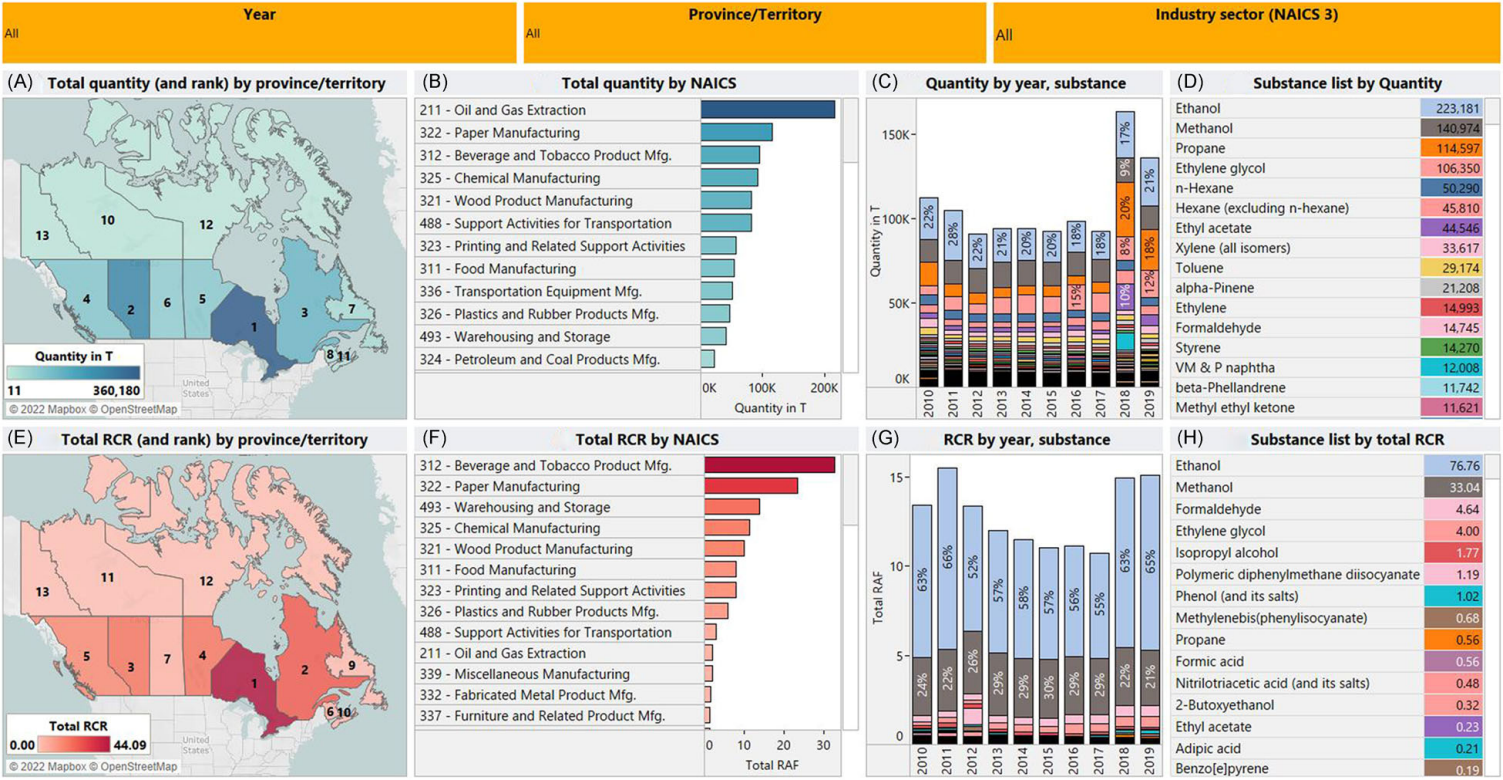
Comparison of the National Pollutant Release Inventory data from 2010–2019 by total quantity and by risk https://public.tableau.com/app/profile/alicia.berthiaume/viz/Figure2_ComparisonofrankingsbyNPRIquantityandbyrisk2010-2019/Figure2?publish=yes

To further illustrate the differences that arise when substances are ranked based on quantity versus risk, several case studies are presented based on two analyses of NPRI data. The first analysis is based on the total quantity released (per province or territory, per year, and per industrial sector) as shown in the top row of Figure 2A–D. The second is based on “total risk” as shown on the bottom row of Figure 2E–H. To estimate “total risk,” we summed the RCRs calculated for each representative organism for each substance by province/territory, by year, and by the industrial sector. The latter perspective assumes additivity of the RCRs and is presented here primarily for illustrative purposes. A more definitive approach would be to examine chemical risk estimates in terms of mode of action. For example, risk estimates are additive for chemicals that exert only a baseline toxicity mode of action, but for chemicals that exert a more selective mode of action, the assumption of additivity is more uncertain.

#### Priority provinces or territories by quantity versus by risk

From the perspective of risk management, understanding where the hot spots of risk are can optimize the use of limited resources to focus on the most pressing issues. To this end, the left‐most panes of Figure 2A, contrast two “hot spot” maps ranking provinces/territories by quantity and by risk. Ontario (ON) is the top priority in 2019 in both cases but not for the same reasons (see Figure S1). High quantities of ethanol released in this province in 2019 are key reasons why it is ranked high by quantity as well as by risk. However, ON also saw the highest releases of formaldehyde, phenol, methylenebis (phenylisocyanate), and isopropyl alcohol. These substances have moderate to high exposure and hazard potential, and combined with their 2019 release volumes, are key reasons why ON ranked as the hot spot province in Canada by risk. Similarly, Saskatchewan (SK) ranks 3^rd^ by quantity in 2019, but because the mixture of substances released is dominated by propane and hexane while other more potent substances are either not present or present only in relatively smaller quantities (e.g., no ethanol, <1 kg of phenol and <10 g of methylenebis [henylisocyanate] were released), SK ranks 8^th^ by risk. The interested reader can visit the dynamic dashboard version of Figure 2 (link in the caption) to explore the results further.

#### Priority sectors by quantity versus by risk

Understanding what industrial sectors reporting to the NPRI program are causing the most concern is another way to allocate finite risk management or regulatory resources to have the most impact on environmental and human health protection. To this end, the priority sectors characterized by North American Industrial Classification System (NAICS), three‐digit codes are examined from both the quantity and the risk perspectives (see Figure 2 and S2b,f). By quantity, the sector defined as NAICS 211—Oil and gas extraction (including and beyond oil sands) appears to be the key priority in 2019, contributing ~28% of the total quantity of 2019 releases mainly to air and water. However, by risk, this sector contributes <3% of the total risk for 2019 because the potency of the major substances emitted by this sector (e.g., propane, all hexane isomers excluding *n*‐hexane) are relatively low in comparison to the mixtures emitted by other sectors. By contrast, NAICS 312—Beverage and Tobacco Product manufacturing (comprising mainly distilleries) contributes about 8% of total releases but is the top sector risk, representing >23% of total RCR, primarily because of very large ethanol emissions to air, which have higher risk potential relative to the large propane and hexane air emissions of the oil and gas extraction sector, among others.

#### Comparison of time trends by different approaches

Examination of trends over time also illustrates differences in the quantity versus risk approaches. For example, the time trend by quantity (Figure 2C and S3) shows an increasing trend over the period of 2010–2019, which is influenced by increases in releases of methanol, ethylene glycol, and other substances to a lesser degree (e.g., ethylene, cyclohexane, etc.), implying that chemical load in the environment is increasing in Canada. However, the time trend by total RCR (Figures 2G and S3) is flat (slope ~0) over the same period, suggesting the level of risk is not increasing. This flat trend can be attributed in large part to changes to the mode of entry of the releases (air, water, and land) over time, and the influence this has on total risk. For example, while methanol releases increased overall, the proportion released to air decreased over time, whereas the proportion released to land and water increased. These land releases of methanol translate to smaller contributions to total risk per tonne than air releases of methanol, so the effect these have on the time trend by risk is muted by the larger downward trend in air releases, resulting in a downward time trend overall for methanol risk. Similarly, increasing quantities of ethylene glycol releases (to land) are noticeable in the quantity time trend, but because they translate to smaller contributions to total risk per tonne than other substances in other environmental compartments (e.g., methanol to air), their impact on the slope of the risk time trend is subtle, and do not equate to a similar rise in risk. Another factor, although minor, is the 2012 spike in releases of methylenebis (phenylisocyanate) and polymeric diphenylmethane diisocyanate, though imperceptible in the quantities time trend, contributed to the anomalously high RCR total for that year. These substances were released in lower quantities since. Overall, it is the change in receiving environmental media of methanol and ethylene glycol releases and the proportionally lower risk that has the greatest influence on the risk time trend, yielding a flat trend.

### Further considerations for ranking using the NPRI and RAIDAR

The contrast in rankings by different approaches described above highlights the need to consider a more holistic view of NPRI data than quantities alone. Doing so will allow a better understanding of progress toward local and planetary health and sustainability and optimize the allocation of risk management efforts to substances and sectors where the most pressing issues can be addressed. However, while this approach to understanding NPRI data in a more holistic manner adds to the knowledge base of potential chemical risks in Canada, it is but one line of evidence in the larger prioritization and risk assessment framework for chemicals in Canada. The Chemicals Management Plan (GOC, 2016a, 2020) is the national framework that considers chemical sources including and beyond those represented in the NPRI, and the results discussed herein should not be taken alone as a final indicator on broad chemical risk. Several known considerations in the application of RAIDAR and the NPRI in the interpretation of risk from emissions estimates are discussed upfront in the introduction, and present future opportunities to improve the accuracy and comprehensiveness of this type of analysis. For example, the default hypothetical regional‐scale environment of RAIDAR can be parameterized to better characterize actual specific regions (e.g., like the ChemCAN model; Kawamoto, et al., 2001), yielding region‐specific simulations. As well, one approach to address non‐NPRI releases is to use the Chemicals in Products—Comprehensive Anthropospheric Fate Estimation (CiP‐CAFE) tool (Li & Wania, 2016) for estimating diffuse chemical emissions to the regional environments. Based on regional‐scale chemical production tonnage information, CiP‐CAFE predicts the rates of emissions to the multiple receiving compartments (air, water, and soil), which can be used to complement NPRI (point source) data and help address data gaps for parameterizing models like RAIDAR for evaluations with monitoring data. Furthermore, by combining CiP‐CAFE and NPRI data, a more complete understanding of exposures and risks at point‐sources and regional‐scales can be determined and potential oversights in using only NPRI data for chemical priority setting can be avoided.

Another opportunity is to address the sizeable NPRI disposals dataset that is not amenable to use in RAIDAR. A scan of organic substances within this dataset (2010–2019) indicates that these total ~510 000 tonnes of disposals, suggesting that further research into the environmental fate and impact of these data merit consideration. Here again, tools like CiP‐CAFE that consider chemical emission factors over the course of a chemical lifecycle may help address this current data gap and uncertainty.

Future work could also address the inorganics excluded here, which comprise the top NPRI substances by release quantity in the last decade and beyond. While models such as the USEtox model (Hauschild et al., 2008) do include some inorganic metals, CACs are not yet included in any known multimedia environmental fate, exposure, and risk estimation models. Future work to address this gap using suitable models for metals and CACs would be valuable to enhance the comprehensiveness of a risk‐based approach for ranking and prioritizing the NPRI. Further justification can be found in the results of a cursory examination of NPRI data from the last five years (as an example) within the USEtox (v2.12) model, which shows that metals, such as zinc, manganese, copper, nickel, mercury, lead, arsenic and cadmium, are among the top priorities when ranking by either human or eco midpoint comparative toxicity units/tonne (Figure S4). Similar analyses using USEtox to rank NPRI substances in Nova Scotia (Taylor et al., 2020) and E‐PRTR data in Sweden (Nordborg et al., 2017) found zinc to pose the most significant human toxicity risk, though Nordborg et al. (2017) recommend future work to reconcile the paradox of zinc being both a priority risk and relatively harmless to humans; a reality in Canada as well.

Future work on targeted monitoring to ground‐truth model predictions can better determine the confidence and uncertainty of combining the NPRI data with these models. This would improve our understanding of chemicals that are currently subject to some monitoring and more importantly perhaps for addressing data gaps for chemicals that are not currently being monitored in the Canadian environment. Future work could also investigate other models more suitable for point‐source emission fate and exposure estimation (for example, AERMOD—American Meteorological Society/Environmental Protection Agency Regulatory Model) to gain more understanding of the impacts of nonsteady dynamics on risk estimations on various geographic scales. Future work to evaluate the potential cumulative effects would improve our understanding of pollutant risks in regions comprising multiple pollutant emissions, which is expected to be the case across Canada and the globe.

## CONCLUSION

PRTRs around the world, including Canada's NPRI, are increasingly being called upon to deliver tools that integrate pollutant release data into more holistic interpretations of risk, impact, and priorities. Doing so contributes to better decision‐making regarding pollutant risk assessment and management at the local to global scale in both ecological and human receptors, and thus enables better understanding and decision‐making in terms of planetary health and sustainability. This study uses the RAIDAR multimedia environmental fate, exposure, and risk estimation to demonstrate proof of concept for discrete organic chemicals on the NPRI. This work integrates toxicity, critical thresholds, physicochemical properties, environmental fate, and exposure simulations and release data for a subset of NPRI organic substances (2010–2019). Results confirm that the magnitude of release alone is inadequate for understanding risk, revealing differences in priority rankings of NPRI organics by quantity alone or by potential risk estimated by RAIDAR. This study advances the body of knowledge on linking NPRI releases to potential risk in both human and ecological receptors; however, these results cannot be taken as standalone indicators of risk and in‐depth risk assessment is needed to draw the most comprehensive and accurate conclusions on local, regional, and national scales. Nonetheless, this approach can be used as a complementary tool to interpret the degree of potential risk associated with NPRI releases both within an in‐depth risk assessment, and more broadly in screening exercises to inform and prioritize effective and timely risk assessment and management planning. More research related to overcoming limitations in both the modeling approach and data available through the NPRI would improve the accuracy and comprehensiveness of future studies of this type. Specifically, future directions could include addressing the inorganic NPRI substances (e.g., CACs and metals), addressing NPRI disposal data, applying more spatially and temporally resolved or point‐source‐specific multimedia mass balance fate and exposure models, improving the accuracy of releases reported to the NPRI, better characterizing sources beyond those captured in the NPRI, and continuing to ground‐truth model predictions against monitoring data to foster confidence and address uncertainty in model predictions.

## CONFLICT OF INTEREST

The authors declare no conflicts of interest.

### OPEN DATA BADGE/OPEN MATERIAL BADGE

This article has earned an Open Data Badge and Open Material Badge for making publicly available the digitally shareable data necessary to reproduce the reported results. The data and material are available at https://open.canada.ca/data/en/dataset/40e01423-7728-429c-ac9d-2954385ccdfb and https://arnotresearch.com/models/. Learn more about the Open Practices badges from the Center for Open Science: https://osf.io/tvyxz/wiki.

## Supporting information

This article contains online‐only Supporting Information.

The Supporting information file contains a description of the RAIDAR model input parameters for NPRI organic substances, a definitions table for NPRI release categories (Table S1), and four figures comparing NPRI pollutant quantities to either RAIDAR or USEtox model outputs (Figures S1–S4).

## Data Availability

All National Pollutant Inventory data used in this study are accessible at the Government of Canada's Open Data portal https://open.canada.ca/data/en/dataset/40e01423-7728-429c-ac9d-2954385ccdfb and/or downloadable from the dynamic visualizations linked in the figure captions. The RAIDAR model is available at: https://arnotresearch.com/models/.
