# Supplementary material for: Risk‐based prioritization of organic substances in the Canadian National Pollutant Release Inventory using an evaluative regional‐scale multimedia mass balance model
Source: Integr Environ Assess Manag. 2022 Apr 5;18(6):1722–32. doi: 10.1002/ieam.4601 (PMC9790719; doi:10.1002/ieam.4601)
Supplement: Supplementary file 1 — The Supporting information file contains a description of the RAIDAR model input parameters for NPRI organic substances, a definitions table for NPRI release categories (Table S1), and four figures comparing NPRI pollutant quantities to either RAIDAR or USEtox model outputs (Figures S1–S4). [file IEAM-18-1722-s001.docx]

Supporting Information -

**Risk-based prioritization of the Canadian National Pollutant Release Inventory using an evaluative regional-scale multimedia mass balance model.**

***Model input parameters for NPRI organic substances***

The main text of this article summarizes a new database of chemical information for 252 NPRI substances required for the RAIDAR model simulations (see Table 1 of main manuscript). Further details in the RAIDAR input parameters and their derivation are presented here.

The new RAIDAR input parameters database for NPRI organic substances includes molar mass (M; g.mol^-1^), the octanol-water partition coefficient (K_OW_; unitless), the air-water partition coefficient (K_AW_; unitless), and the acid dissociation constant (pKa) for ionizable organic chemicals (IOCs) only. The octanol-air partition coefficient (K_OA_) is also used in the model. Following the “three solubility approach”, K_OA_ is calculated within RAIDAR code from K_OW_ and K_AW_ as K_OA_ = K_OW_/K_AW_ (Cole and Mackay 2009). For IOCs the partition coefficients and physical-chemical properties are for the neutral form of the chemical, i.e., K_OW,N_ and K_AW,N_. For IOCs, the dissociation constant (e.g. pKa) and the apparent partition coefficients for the charged species (e.g., K_OW,I_ K_OC,I_) can also be included as input parameters, if these data are available. If K_OW,I_ and K_OC,I_ data are not available, the model will assume and calculate default values based on the neutral form and a default scaling factor (Toose et al. 2004). RAIDAR can only accept a single dissociation constant for an IOC and the IOC is determined to be either an acid or a base. Molecules with complex and multiple ionization centers and zwitterions are simulated using the dissociation constant of the most acidic or basic ionogenic functional group. It is emphasized that this is a necessary simplifying assumption and that many IOCs may have multiple ionization centers containing both acidic and basic groups.

The physical-chemical properties relate directly to molecular structure. All RAIDAR simulations require chemical-specific information including identification (Chemical Abstract Services (CAS) #, name, or Simplified Molecular-Input Line-Entry System (SMILES) (Weininger 1998), physical-chemical property data (internally consistent partition coefficients) and degradation half-lives in air, water, soil, sediment, and biotransformation half-lives for vertebrates (fish, birds, mammals). Structural information (SMILES) for the discrete organic moiety was obtained from the NPRI CAS RNs using EPI Suite™ Ver. 4.11 (US EPA 2012). SMILES were then de-salted and canonicalized using Open Babel (O'Boyle et al. 2011) and these SMILES were used for obtaining physical-chemical property and degradation half-life data.

The US EPA’s EPI Suite™ Ver. 4.11 was used as the primary source for obtaining physical-chemical properties. Measured values are selected preferentially over predicted values. The pKas for the IOCs were obtained from ACD Labs Percepta using the Classic pKa algorithms. ACD Labs identifies the major base (MB), the major acid (MA) as well as other base (B) and acid (A) ionization centers. MB and MA are pKas associated with highest and lowest pKas, respectively, thus these pKas are expected to largely characterize the primary charges on the IOC.

RAIDAR requires degradation half-life data for air, water, soil and sediment compartments and biotransformation half-lives in vertebrate species, i.e., fish, birds, and mammals. Half-lives in air were estimated for ozone and hydroxyl radical reactions using AOPWIN in EPI Suite. Empirical reaction rates based on ozone and hydroxyl radicals were selected preferentially over QSAR predicted rates. The default assumptions for radical concentrations were assumed (5×10^5^ radicals/cm^3^ and 7.0×10^11^ mol/cm^3^, respectively) and a 24-hour reaction period was assumed. When relevant, chemical half-lives in air were estimated by reciprocally combining the AOPWIN hydroxyl radical and ozone half-lives. Atmospheric degradation in the environment was assumed to only occur in the gas phase (i.e., particle bound fraction of chemical was assumed to be unavailable for degradation). For chemicals for which degradation half-lives in air could not be calculated, the half-lives in air were assumed to be 10^6^ h; highly persistent (conservative).

Estimates of aerobic aqueous phase biodegradation half-lives were obtained using a method that calibrates BIOWIN model outputs from EPI Suite to empirical environmental biodegradation half-lives (Arnot et al., 2005; Arnot et al., 2012). The BIOHCWIN model was also used to estimate half-lives for hydrocarbons on the NPRI list and these predictions were selected preferentially over the predictions derived from the BIOWIN calibration method. Biodegradation reactions were assumed to occur in the bulk water compartment, i.e., dissolved and particle bound. Hydrolysis reactions are often very sensitive to pH which can be quite variable in the environment. We did not consider degradation reactions resulting from hydrolysis and photolysis largely because such data are not available; however, these could be important degradation processes for certain chemicals. Half-lives in soil and sediment followed the extrapolation ratio of 1:2:9 from water, i.e. water:soil:sediment (Aronson et al. 2006). No anaerobic degradation was considered in these simulations as the “active” sediment compartment in which the organisms live is assumed to be primarily aerobic. Biotransformation half-lives in fish were obtained using the fish biotransformation half-life QSAR in the BCFBAF model in EPI Suite (Arnot et al., 2009) and the IFS-QSAR (Brown et al., 2012). The geometric means were calculated from the model predictions. Biotransformation half-lives in mammals and birds were obtained using the human biotransformation half-life IFS-QSAR (Arnot et al. 2014). Empirical biotransformation half-life data were selected preferentially to QSAR predictions. In the RAIDAR model biotransformation half-lives are scaled for differences in species-specific body size using allometric equations.

Toxicity or effects/no effects data can be entered into RAIDAR in various ways to parameterize the model for HAF, RAF and EC calculations. Internal effect concentration (IEC, or critical body residue; CBR) data or L(E)C50 for chemical toxicity can be used. Currently, the L(E)C50 can be entered for the general taxonomic categories of plants/algae, invertebrates, and fish. For birds and mammals IECs can be used or an intake rate corresponding with an effect or no effect intake rate (i.e., TTC).

The primary source of ecotoxicity data for aquatic species was the EnviroTox database developed by multiple stakeholders through a recently completed Human and Environmental Sciences Institute project (Connors et al., 2019). Two primary advantages of this database are that it has been developed from most other publicly available ecotoxicity databases (e.g., initially ~220,000 records from data sources like US EPA’s ECOTOX database (2021) and the European Chemicals Agency (ECHA)’s Registration, Evaluation, Authorisation and Restriction of Chemicals (REACH) program) and that it has been curated to some degree for structural and chemical identification reliability. Of the 252 NPRI CAS RNs, 166 were found in the EnviroTox database. To the greatest extent possible, taxa-specific effect concentrations (e.g., LC50s for fish, EC50s for invertebrates and algae) were used to parameterize RAIDAR. For chemicals on the NPRI list without EnviroTox data, 28 chemicals had toxicity data used in recent Environment and Climate Change Canada (ECCC) Domestic Substances List (DSL) work (ECCC, 2016). For the remaining 58 chemicals without ecotoxicity data in EnviroTox or ECCC DSL sources, the US EPA T.E.S.T. modelling software was used (Martin 2016). Experimental data from T.E.S.T. were selected preferentially over QSAR predictions. There were four chemicals with no toxicity predictions available. For the aquatic species, these chemicals were assumed to be baseline toxicants with an internal effect concentration of 3 mmol/kg-ww.

There is a paucity of ecotoxicity data for avian and mammalian species. To address this, data used in developing the TTC approach were used to parameterize a “no effect” intake rate for birds and mammals in these simulations. The case study simulations use a threshold for no effects for birds and mammals of 0.15 mg/kg/d which corresponds to fifth percentiles from log normal cumulative distributions of NOELs (Munro 1996). Following this approach, the toxicity assessment endpoints between aquatic and air-breathing species are not consistent (e.g., apical effects vs. no effects, respectively); however, this approach adequately provides proof of concept for the application of the model and initial values for NPRI evaluations and deliberations. Table 1 of the main manuscript also provides an estimate of the rate of chemical emissions, based on the NPRI release data (to air, water, and land) reported in recent reporting years (2010-2019) used to parameterize the model for the current applications.

**Table S1:** NPRI definitions of release categories (ECCC, 2020):

| **Releases:**  A discharge of a substance to the environment within the physical boundaries of the facility. This includes releases to air, surface waters and land. Routine and accidental/non-routine releases (viz. spills) are included, but disposals (on- and off-site) and transfers for recycling (off-site) are not. | **To air** | **Stack or point:** releases from stack or point sources including stacks, flares, vents, ducts, pipes, other confined process streams, and from pollution control equipment. |
| --- | --- | --- |
|  |  | **Storage or handling:** releases to air from storage or handling of materials. |
|  |  | **Fugitive:** releases that cannot be captured and/or that are unintentional, including equipment leaks (form valves, pumps seals, flanges, compressors, sampling connections, open-ended lines, etc.), evaporative losses from surface impoundments and spills, releases from building ventilation systems, and any other fugitive or non-point air emissions from land treatment, tailings, waste rock, storage piles, etc. |
|  |  | **Spills:** accidental releases to air |
|  |  | **Road dust:** total particulate matter, PM10 and PM2.5 releases from road dust (if vehicle travelled >10,000km on unpaved roads at the facility) |
|  |  | **Other non-point sources:** any other non-point releases to air not captured in the categories above. |
|  | **To surface waters** (including oceans, lakes, rivers, streams, wetlands, etc.) | **Direct discharges:** releases directly discharged to surface waters from within the site boundary, including those from onsite wastewater treatment systems or sewers that leave the facility and do not feed into an off-site wastewater treatment plant Discharges feeding onto an off-site wastewater treatment facility are not included here, and are included as off-site transfers (for treatment prior to final disposal). |
|  |  | **Spills:** includes any short-term accidental releases (e.g. hours to days) |
|  |  | **Leaks:** includes any long-term/chronic accidental releases (e.g. weeks to months, etc.) |
|  | **To land** (including surface and underground releases at a facility) | **Spills:** includes any short-term accidental releases (e.g. hours to days) |
|  |  | **Leaks:** includes any long-term/chronic accidental releases (e.g. weeks to months, etc.) |
|  |  | **Other releases to land:** Includes net quantities released to land (that are not disposals) not captured in the categories above, such as underground injections for purposes other than disposal (e.g. solvents used for in-situ bitumen extraction). Discharges to land for purposes of disposal are excluded here, but included as disposals (various categories). |

FIGUREs


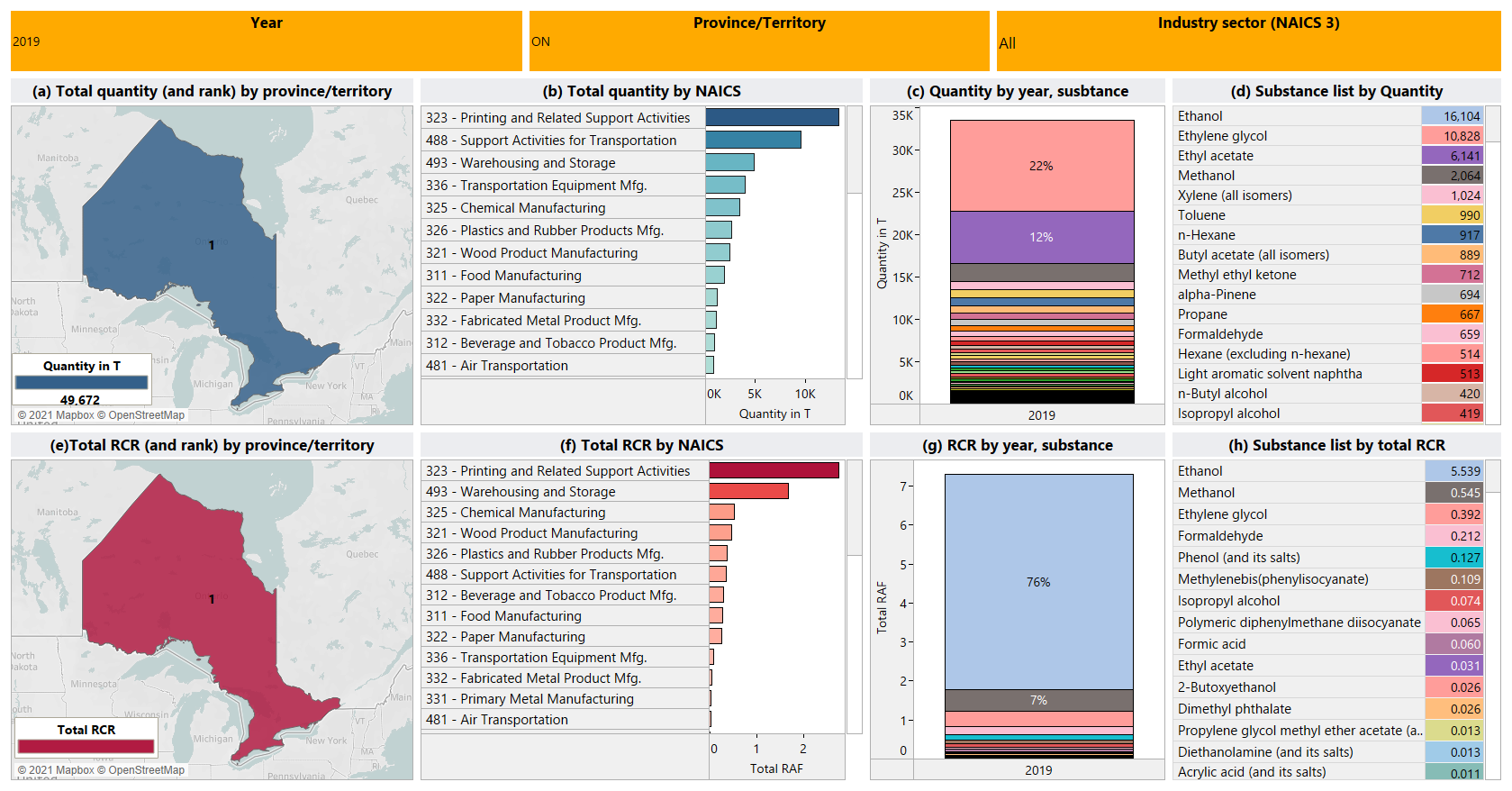


**Figure S1**: A view of Ontario priorities in 2019, by quantity in tonnes (top row, panes a - d) and by risk (bottom row, panes e - h) showing that while Ontario is the top priority in Canada by both perspectives, but not for the same reasons.


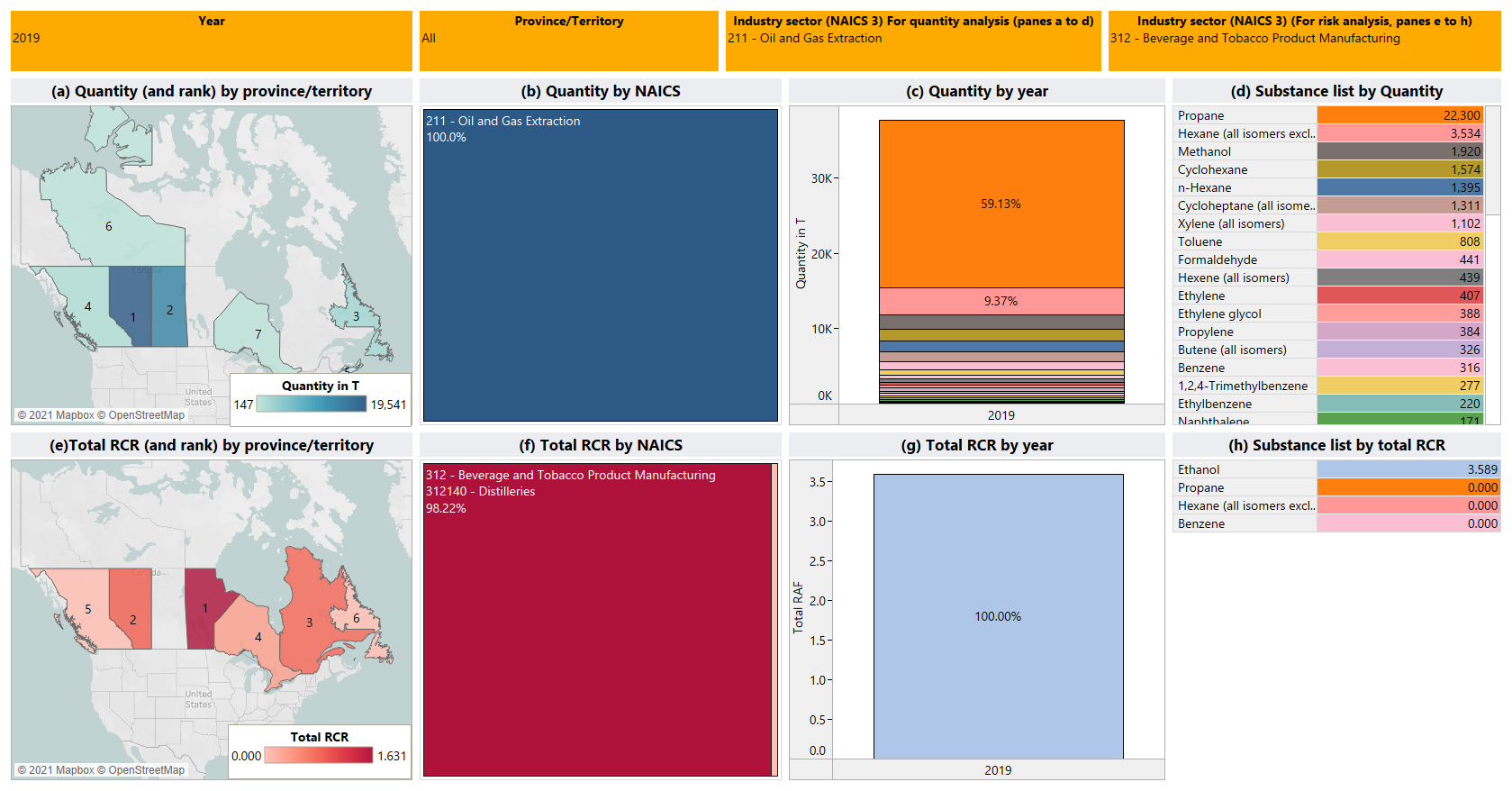


**Figure S2:** A filtered view focusing on the top industrial sector priority in 2019 by quantity in tonnes (top row, panes a – d, NAICS 211 – Oil and gas extraction), and by risk (bottom row, panes e – h, NAICS 312 – Beverage and tobacco product manufacturing). This focused view shows that the substances driving the results for each perspective differ; large ethylene glycol releases push the “Support activities for air transportation” sector to the top priority by quantity, whereas by risk, the Chemical pulp mills sector is the top priority, driven mainly by the risk due to releases of methanol.


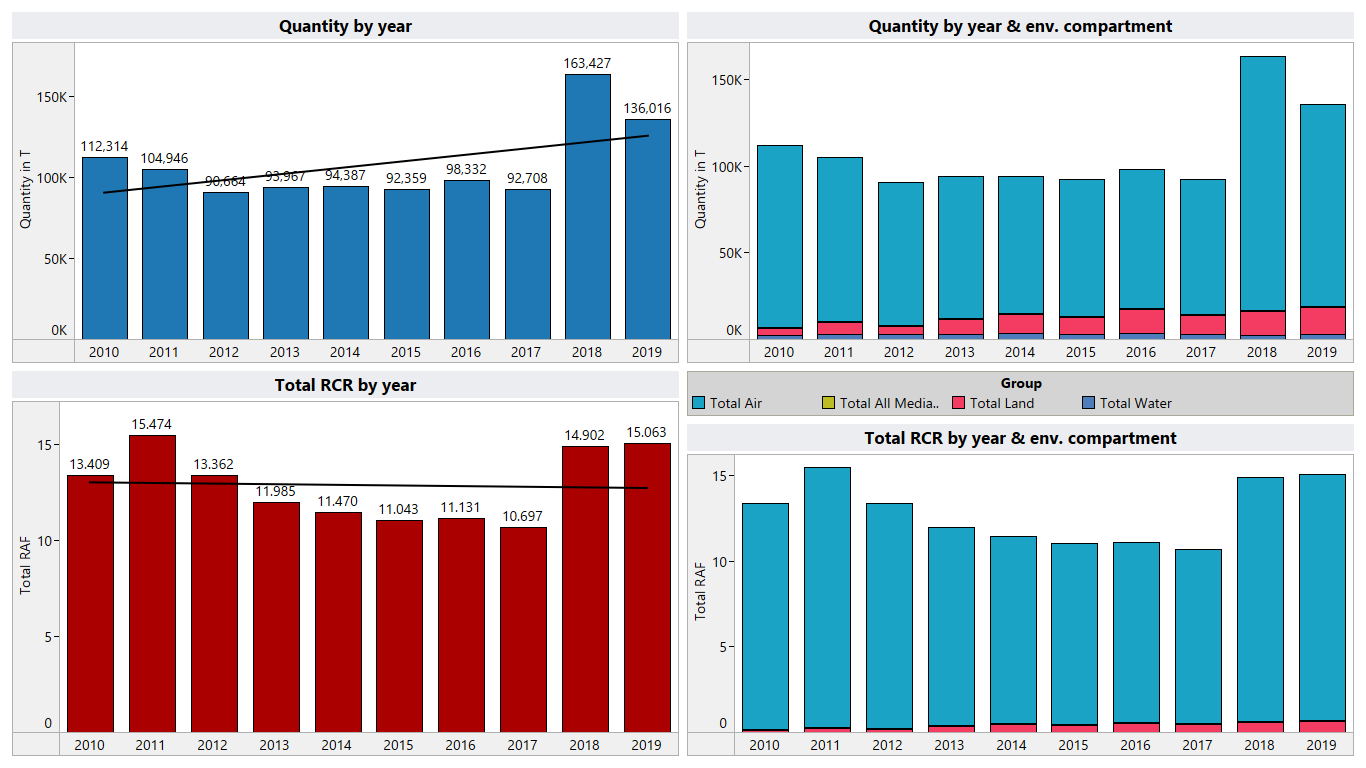


**Figure S3**: A view of the time trends in totals by quantity in tonnes (top row: left is total, right is the same graph but colour-coded by environmental compartment of the release) and risk (bottom row: left is total, right is the same graph but colour-coded by environmental compartment of the release) over 2010-2019.

**Figure *S*4:** Comparison and trends (2015-2019) of NPRI release quantities for the subset of substances with corresponding USEtox v2.12 (2019) eco or human toxicity characterization factors with resulting comparative eco and human comparative toxicity units. USEtox midpoint characterization factors were multiplied by the corresponding media-specific NPRI release quantities.


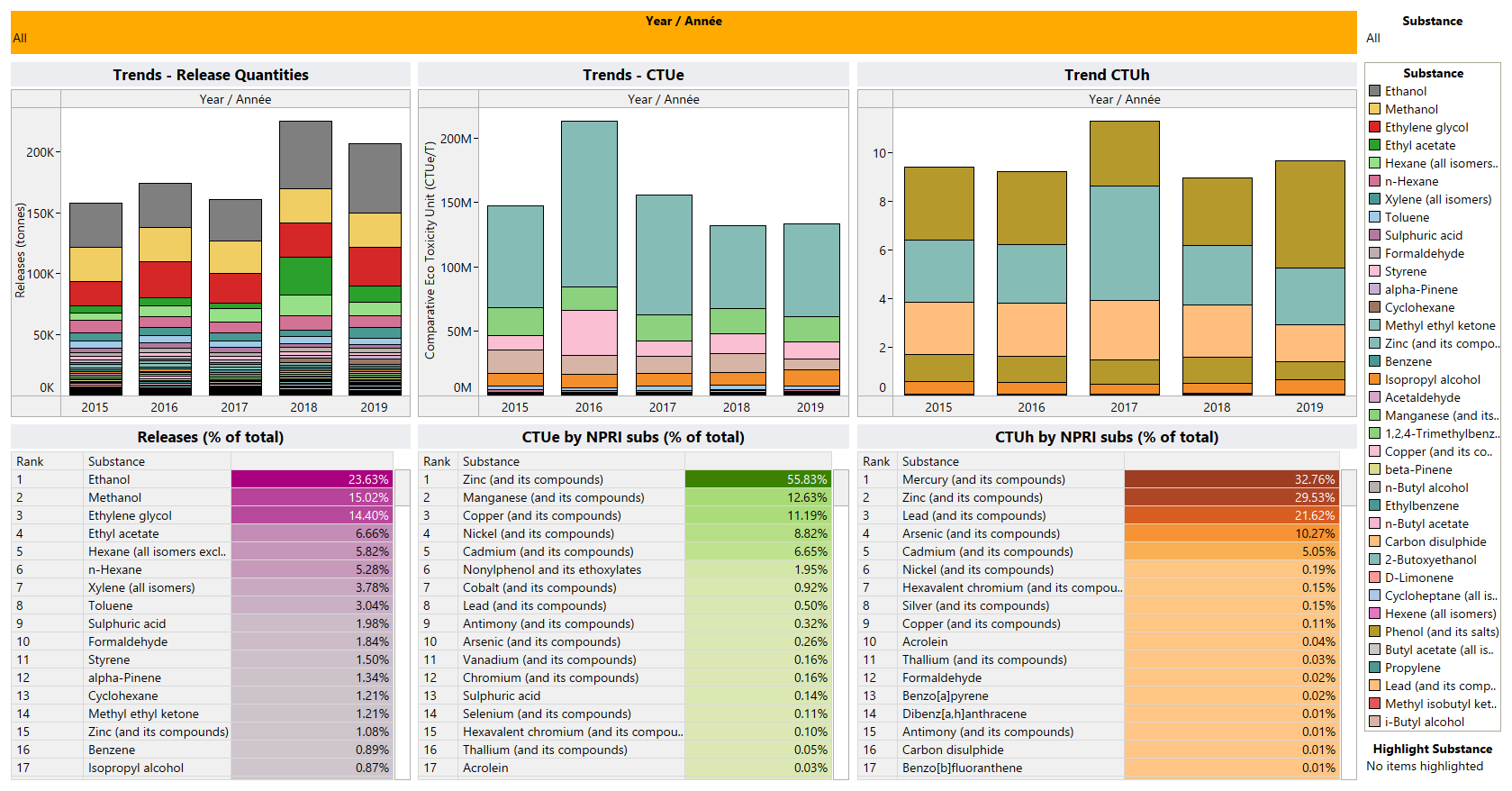


**References**

Arnot JA, Gouin T, Mackay D. 2005. Practical methods for estimating environmental biodegradation rates. Report for Environment Canada. Peterborough, ON: Canadian Environmental Modelling Network, Trent University. No. CEMN2005003.

Arnot JA, Meylan W, Tunkel J, Howard PH, Mackay D, Bonnell M, Boethling RS. 2009. A quantitative structure-activity relationship for predicting metabolic biotransformation rates for organic chemicals in fish. Environmental Toxicology and Chemistry. 28(6):1168-1177.

Arnot J, Brown T, Wania F, Breivik K, McLachlan M. 2012. Prioritizing chemicals and data requirements for screening-level exposure and risk assessment. *Environ. Health Perspect., 120*12, 1565-1570. doi:10.1289/ehp.1205355

Arnot J, Brown T, Wania F. 2014. Estimating screening-level organic chemical half-lives in humans. *Environ. Sci. Technol., 48*, 723-730. doi:10.1021/es4029414

Aronson D, Boethling R, Howard P, Stiteler W. 2006. Estimating biodegradation half lives for use in chemical screening. *Chemosphere*, 1953-1960. doi:10.1016/j.chemosphere.2005.09.044

Brown TN, Arnot JA, Wania F. 2012. Iterative fragment selection: A group contribution approach to predicting fish biotransformation half-lives. Environmental Science and Technology. 46:8253-8260.

Cole J, Mackay D. 2009. Correlation of environmental partitioning properties of organic compounds: The three solubilities approach. *Environ. Toxicol. Chem., 19*, 265-270. doi:10.1002/etc.5620190203

Connors K, Beasley A, Barron M, Belanger S, Bonnell M, Brill JE 2019. Creation of a curated aquatic toxicology database: Envirotox. *Environ. Toxicol. Chem., 38*5, 1062-1073. doi:10.1002/etc.4382

[ECCC] Environment and Climate Change Canada. 2016. Science approach document: Ecological risk classification of organic substances. Gatineau: Environment and Climate Change Canada. Retrieved February 25, 2021, from https://www.ec.gc.ca/ese-ees/A96E2E98-2A04-40C8-9EDC-08A6DFF235F7/CMP3%20ERC_EN.pdf

[ECCC] Environment and Climate Change Canada. 2020. Guide for reporting to the National Pollutant Release Inventory. Available from: <http://publications.gc.ca/collections/collection_2020/eccc/En81-1-2020-eng.pdf> [Accessed 4 March 2021]

Martin T. 2016. Toxicity estimation software tool TEST. Washington, D.C. Retrieved from <https://www.epa.gov/chemical-research/toxicity-estimation-software-tool-test>

Munro I, Ford R, Keenpohl E, Sprenger JG. 1996. Correlation of structural class with no-observed-effect levels: A proposal for establishing a threshold of concern. *Food Chem. Toxicol., 9*, 829-867. doi:10.1016/s0278-69159600049-x

O'Boyle N, Banck M, James C, Morley C, Vandermeersch T, Hutchison G. 2011. Open babel: An open chemical toolbox. *J. Cheminformatics, 3*, 33. doi:10.1186/1758-2946-3-33

Toose L, MacKay D. 2004. Adaptation of fugacity models to treat speciating chemicals with constant species concentration ratios. *Environ. Sci. Technol., 38*, 4619-4626. doi:10.1021/es049957i

[US EPA] United States Environmental Protection Agency. 2012. Estimation programs interface epi suite for microsoft® windows, ver. 4.11. Washington, D.C.: United States Environmental Protection Agency.

[US EPA] United States Environmental Protection Agency. 2021. ECOTOX Knowledgebase. Retrieved 09 22, 2021, from United State Environmental Protection Agency: https://cfpub.epa.gov/ecotox/

USETox 2.12. (2019). USEtox. Retrieved from <https://usetox.org/> [Accessed 4 March 2021]

Weininger D. 1988. Smiles, a chemical language and information-system.1. Introduction to methodology and encoding rules. *J. Chem. Inf. Comput. Sci., 28*, 31-36. doi:10.1021/ci00057a005
